# The Opportunity for Impactful Integration of Vascular and Podiatric Care

**DOI:** 10.3390/jcm12196237

**Published:** 2023-09-27

**Authors:** Young Kim, Kevin W. Southerland

**Affiliations:** Division of Vascular and Endovascular Surgery, Duke University, Durham, NC 27708, USA; kevin.southerland@duke.edu

**Keywords:** valuation, vascular surgery, podiatry, limb salvage

## Abstract

Background: The importance of collaboration between vascular and podiatric surgeons has been well-established. High-level partnerships are integral to the development of multidisciplinary programs and wound care centers, ultimately resulting in improved patient outcomes. This vascular–podiatric integration is not universal, however, and podiatric surgery may not be aligned within a vascular surgery division at many institutions. As one such institution, we reviewed our single-center experience in order to identify opportunities for the impactful integration of vascular–podiatric patient care. Methods: Institutional electronic medical records were retrospectively reviewed for all procedures performed by vascular surgeons at a high volume, safety-net academic medical center. Data were collected on all primary and additional procedures, current procedural terminology (CPT) codes, case type (elective, urgent, emergent), surgeon specialty, and date/time of the procedures performed. CPT codes were linked to the Centers for Medicare & Medicaid Services’ Physician Fee Schedule to estimate the work relative value unit (wRVU) per procedure. Results: From 2018 to 2022, vascular surgeons performed a total of 12,206 operations, of which 1102 (9.9%) involved podiatric procedures. The most common vascular-performed podiatry procedures performed were toe amputations (38.1%, n = 420), transmetatarsal foot amputations (20.1%, n = 222), and ankle/foot debridement (16.2%, n = 178). Foot/ankle-specific procedures were identified as the primary procedure in 726 (65.9%) cases and as the adjunct procedure in 376 (34.1%) cases. A substantial proportion of podiatric procedures occurred on an urgent (n = 278, 25.2%) or emergent (n = 28, 2.5%) basis. A total of 163 (14.8%) cases occurred after hours (either before 0600 or after 1800), and 133 (12.1%) cases were performed on a holiday or weekend. Procedure-specific revenue included 4243.39 wRVU for primary procedures and 2108.08 wRVU for additional procedures performed. Conclusions: We report our single-center experience in which vascular surgeons provide a significant proportion of podiatric procedures. Our study underscores the potential for integrating podiatric surgeons within a vascular surgical division and presents opportunities for collaboration and enhanced patient care.

## 1. Introduction

Podiatrists play a crucial role in the prevention and treatment of foot wounds in diabetic patients, a fact underscored by a wealth of research findings [1,2,3,4]. This is especially pertinent given the alarmingly high prevalence of diabetic foot-related issues across the United States [5,6], which not only have significant health implications but also substantial economic repercussions in terms of healthcare expenditure [7]. To become proficient in their field, podiatrists undergo a rigorous and comprehensive training regimen in foot and ankle pathologies, including four years of doctoral-level education, followed by three years of surgical residency. Furthermore, many podiatrists opt for additional fellowship-level training to hone their expertise even further [2,8,9]. This extensive training equips them with an intricate understanding of foot anatomy, biomechanics, and surgical skills, allowing them to effectively manage a wide array of foot and ankle diseases with precision and expertise.

The connection between diabetic foot wounds and vascular disease is widely acknowledged within the medical community, supported by a substantial body of research and clinical observations [10,11,12]. Hence, fostering collaboration between vascular and podiatric surgeons is of paramount importance [13]. Strong partnerships between these specialties are essential for the development of effective multidisciplinary programs and specialized wound care centers [14,15,16]. Such collaborations have demonstrated significant reductions in major amputations and improved overall patient outcomes [14,17,18,19]. However, it is important to acknowledge that the integration of podiatric surgery within a vascular surgical division is not a universally implemented practice across all healthcare institutions. This divergence in approach can lead to potential challenges, including competing interests, as it pertains to service coverage, call schedules, and financial incentives.

In light of this, our institution took the initiative to scrutinize our single-center experience in order to identify opportunities for the meaningful integration of vascular –podiatric patient care. Our hypothesis was centered around the idea that when podiatrists are not fully integrated within a vascular surgical division, vascular surgeons may find themselves shouldering additional responsibilities, such as podiatric consultations, surgical interventions, postoperative care, and long-term follow-up. Our primary focus was on assessing podiatric volume and case-mix performed by vascular surgeons, seeking to uncover valuable insights that could inform and potentially enhance our healthcare practices.

## 2. Methods

### 2.1. Data Collected

Institutional electronic medical records (EMRs) were retrospectively reviewed for all procedures performed by vascular surgeons at a large, tertiary academic medical center. Specifically, we utilized the SlicerDicer function of the Epic^®^ EMR system, which is used to review all cases performed over the study period. The “All Invasive Procedures” function was used to query surgical procedures, which were further subdivided based on the operating surgeon. We included all vascular surgeons that were employed by our hospital system over the five-year period, which included surgeons who have since left the practice. All procedures were included in which the vascular surgeon was the primary surgeon for the operation performed. The exclusion criteria included any procedures in which a podiatric surgeon was involved as a co-surgeon or secondary surgeon for any of the operations. There were no other exclusion criteria based on patient age, demographics, or comorbidities, which were not collected for this study.

Our study included two of the three hospitals, with vascular surgery coverage from our group. Procedural data from the regional Veterans Affairs Medical Center (VAMC) were not collected, as the VAMC utilizes a separate EMR system which was not available for review. The five-year study period was from January 2018 to December 2022.

### 2.2. Variables Defined

Data were collected on all primary and additional procedures, current procedural terminology (CPT) codes, case type, surgeon specialty, and date/time of the procedures performed. Case types were categorized into elective, urgent, and emergent cases. After-hours cases are defined as procedures with start times before 0600 or after 1800. Patient demographic data were not collected, as this was outside of the scope of this study.

To determine which cases involved podiatry procedures, search strings of “foot” or “ankle” or “toe” were utilized. Selected cases were then each manually reviewed for inclusion. Specific CPT codes meeting the inclusion criteria and their corresponding procedures were 11010 (debridement at site of open fracture/dislocation, foot), 11012 (debridement at site of open fracture/dislocation, leg), 11042 (debridement, subcutaneous tissue, ankle/foot/toe), 11043 (debridement, muscle and/or fascia, ankle/foot/toe), 11044 (debridement, bone, ankle/foot/toe), 11061 (incision and drainage of abscess, ankle/foot/toe), 10180 (incision and drainage of complex postoperative wound infection, ankle/foot/toe), 11010 (debridement at site of open fracture/dislocation, foot), 13160 (secondary closure of surgical wound, ankle/foot/toe), 15100 (split-thickness skin graft, leg), 15120 (split-thickness skin graft, ankle/foot/toe), 28800 (foot amputation, midtarsal), 28805 (foot amputation, transmetatarsal), 28808 (fasciotomy, foot and/or toe), 28810 (toe amputation, metatarsal), 28820 (toe amputation, metatarsophalangeal joint), and 28825 (toe amputation, interphalangeal joint).

All major amputations (i.e., above-knee amputation, below-knee amputation, any guillotine amputations above the ankle) were not categorized as podiatry procedures. All CPT codes were queried in the Centers for Medicare & Medicaid Services’ Physician Fee Schedule database in order to calculate the work relative value unit (wRVU) per procedure [20]. These data were de-identified per protocol and exported from the SlicerDicer function of the Epic^®^ EMR system into a password-protected spreadsheet for statistical analysis.

### 2.3. Statistical Analysis

All statistics are reported as the frequency (n) and percentage (%) where appropriate. No statistical tests were performed given that there were no comparisons to be made, either univariate or multivariable. Statistical significance was not defined for this study given the lack of comparisons. Statistical calculations were performed using the software package JMP Pro version 16.1 (SAS Institute Inc., Cary, NC, USA). This study was determined exempt by the Institutional Review Board (IRB) at Duke University (IRB number Pro00113208).

## 3. Results

### 3.1. Foot/Ankle Procedures Performed by Vascular Surgeons

Vascular surgeons performed a total of 12,206 operations over the five-year study period. Upon further analysis, a total of 1102 (9.9%) involved a podiatry procedure, either as a primary or additional case. The remaining 11,104 operations (90.1%) were vascular-specific procedures. This podiatric operative volume increased annually, starting at 148 procedures in 2018, and ending at 233 procedures in 2022. Primary podiatric procedures numbered 726 cases (65.9%), outnumbering the adjunctive procedures performed in 376 total cases (34.1%). These details are summarized in Table 1.

### 3.2. Timing of Podiatric Procedures Performed

The majority of these procedures were elective in nature, numbering 796 cases performed (72.2%). There were, however, a substantial proportion of podiatric procedures which were performed on a more urgent (n = 278, 25.2%) or emergent (n = 28, 2.5%) basis. Notably, a total of 163 (14.8%) cases occurred after-hours (as defined as a start time before 0600 or after 1800), and an additional 133 (12.1%) cases were performed on a holiday or weekend. The most common weekday was Friday (n = 227, 20.6%). The timing and class of the podiatric procedures are reported in Table 2.

### 3.3. Details of Specific Podiatric Procedures Performed

Table 3 details all podiatry procedures performed by vascular surgeons as singular, primary procedures. Each CPT code included is reported, along with the corresponding procedure. These are procedures that were not performed in conjunction with other vascular procedures, such as endovascular intervention or open bypass procedures. The most common performed primary (solo) procedures included transmetatarsal amputation (TMA) (n = 173, 23.8%), muscle/fascia debridement of the foot/ankle (n = 141, 19.4%), and metatarsophalangeal toe amputation (n = 139, 19.1%). Primary procedures generated a total of 4243.39 procedure-related wRVUs over the five-year study period.

Table 4 details all podiatry procedures performed by vascular surgeons as additional procedures. These are procedures that occurred in conjunction with other, vascular interventions. The CPT codes and corresponding procedures are detailed below. The most common adjunct procedures included metatarsophalangeal toe amputation (n = 83, 22.1%), metatarsal toe amputation (n = 80, 21.3%), and TMA (n = 65, 17.3%). These procedures generated an additional 2108.08 procedure-related wRVU over the same time-frame. 

## 4. Discussion

In this comprehensive study, we undertook a meticulous review of our institution’s single-center experience, with a particular focus on the unique aspect of vascular surgeons assuming a substantial role in the provision of podiatric care. The scope of our investigation was extensive, encompassing a diverse array of foot and ankle procedures conducted over the course of the study period. Notably, our findings revealed that vascular surgeons performed over 1000 intricate foot and ankle operations during this period. These procedures spanned a wide spectrum of procedures, including toe amputations, TMAs, surgical debridement, skin grafting, and an array of other intricate foot and ankle operations.

Delving deeper into the data, it becomes evident that a significant proportion of these operations were carried out as the sole and primary procedure. This underscores our hypothesis that vascular surgeons are not only performing podiatric procedures in conjunction with revascularization operations but also solo podiatric procedures themselves. Additionally, it should be emphasized that these services encompassed not only surgical interventions but also vital aspects such as consultations, postoperative care, and the critical long-term follow-up necessary to ensure optimal patient outcomes. Taken collectively, these robust data paint a compelling picture that highlights the pivotal role played by vascular surgeons in delivering podiatric services in the absence of full integration with podiatrists. 

The economic implications of the strategic integration of a dedicated podiatric division within the field of vascular surgery have been a subject of recent study and were meticulously detailed in a research effort by Patel and colleagues in 2022 [21]. The scope of their investigation spanned a three-year period, during which their multidisciplinary team saw notable expansion, growing from a team of two vascular surgeons to an impressive cadre of four, while the podiatric component of their team similarly flourished, increasing from a single podiatrist to a robust team of four highly skilled specialists. Throughout this period, a total of over 5000 procedures were performed, collectively encompassing a wide spectrum of both vascular and podiatric operations. Of particular note is the substantial contribution made by podiatrists, who played a pivotal role in this multidisciplinary endeavor. In fact, they contributed a remarkable 40% of the total wRVU to the limb salvage program, a statistic that underscores the indispensable nature of their expertise in the overall success of the program.

The economic implications of this integration are compelling. Patel et al. reported a consistent annual growth in the hospital’s contribution margin for each year under scrutiny. This financial metric is a testament to the program’s ability to not only sustain but indeed bolster the hospital’s financial outlook. The data provided in their study unequivocally demonstrate the tangible financial value derived from the seamless integration of podiatric and vascular services within a comprehensive multidisciplinary limb salvage program.

Over the course of the past decade, the concept of multidisciplinary “toe and flow” programs has witnessed a remarkable surge in popularity within the medical community, driven by collaborative efforts spearheaded by both podiatrists and vascular surgeons alike [16,22,23]. This noteworthy trend underscores the growing recognition of the benefits that can be reaped from fostering high-level partnerships between these surgical subspecialties. These alliances have consistently yielded substantial improvements in patient outcomes and notable reductions in major amputations, thus validating their effectiveness.

However, it remains important to acknowledge that while the concept of integrating vascular surgery and podiatry has made impressive strides, it is not a universally embraced practice across all healthcare institutions. In some instances, podiatry may find itself aligned within different hospital departments, such as orthopedic surgery [24], thereby giving rise to a potentially intricate landscape of overlapping interests in the provision of foot and ankle services [25,26,27,28]. Consequently, there may be potential challenges regarding clinical practice patterns, consultations and coverage, call schedules, and financial incentives. 

Our institution embarked on a comprehensive investigation to explore the possibilities surrounding the integration of podiatric surgeons within the realm of a vascular surgical division. The extent of this exploration was extensive, with vascular surgeons emerging as the primary operators in over 1000 podiatric procedures performed over the study period. Notably, nearly a third of these procedures were performed under urgent or emergent circumstances, underscoring the time-sensitive nature of these interventions. Equally noteworthy is the fact that 12% of these procedures were conducted during holidays or weekends, with an additional 15% requiring the dedication of after-hours resources.

Beyond the temporal considerations, our investigation delved into the financial aspect of providing podiatry services within the vascular division. The cumulative procedural wRVUs exceeded 4000 for primary procedures, with an additional 2000 wRVUs attributed to adjunct procedures. It is important to note that these wRVUs were calculated exclusively for surgical interventions, thus not encompassing the contributions made by nonoperative consultations, bedside interventions, clinic visits, and long-term wound care services. Consequently, when one considers the totality of clinical productivity, it becomes evident that the actual impact of integrating podiatric surgeons is likely even more significant than what is reflected by these numerical values alone. These findings offer a multifaceted perspective on the potential advantages and the broader implications of incorporating podiatry services within a vascular surgical division, providing a nuanced insight into the multifaceted landscape of modern healthcare delivery. 

In the present study, we also discussed investigating postoperative outcomes after podiatric procedures and comparing the two specialties. However, we had some difficulty in finding the appropriate outcomes for comparison. Specifically, we considered five separate outcomes and found significant limitations in each. The first outcome was surgical site infection. Many of these podiatric procedures are performed for the indication of wet gangrene or osteomyelitis, however, which vastly increases the possibility of developing wound infection. The second outcome was unplanned return to the operating room. This is often the case in patients with residual infection at the proximal margin of amputation or need for continued debridement, rather than a reflection of the surgeon’s specialty. The third outcome was ambulatory status. Unfortunately, many of these patients are non-ambulatory or have limited ambulation at the time of presentation. The fourth outcome was the hospital length of stay. This outcome would be limited by patient comorbidities and social factors, such as their insurance status, which ultimately determine discharge disposition and placement. The fifth and final outcomes were major systemic complications and postoperative mortality. These outcomes are unlikely to result from the podiatric procedure itself, which is frequently performed under local or regional anesthesia, and more likely a consequence of patient-related comorbidities. Given each of these limitations, we ultimately elected not to compare postoperative outcomes in this study.

Despite these limitations in reporting the outcomes, three other studies have reported their outcomes after developing a streamlined, multidisciplinary diabetic foot care service. Each series reported either a change in the amputation profile or an overall reduction in major amputations following multidisciplinary integration. In 2004, one tertiary care center in Turkey implemented a diabetic foot care team involving podiatrists, infectious disease specialists, endocrinologists, plastic/reconstructive surgeons, and diabetic foot nurses, amongst others [17]. In their retrospective series of 66 patients with diabetic foot ulcers, the authors reported a change in amputation profiles, with a slight decrease in major amputation rates following implementation. Ultimately, any peripheral vascular disease, gangrene, or osteomyelitis were found to be predictors of amputation in this population. In 2009, Hedetoft et al. reported similar findings from their single-center series in Denmark [18]. A total of 88 patients with diabetic foot ulcers were reviewed over a six-year period after implementation of a multidisciplinary diabetic foot clinic. Despite a four-fold increase in foot ulcers encountered over the time period, there was no significant increase in major amputation rates, suggesting that this may be due to improved care provided by the integrated team. Also, in 2009, a separate tertiary care center in Turkey reported a reduction in amputation rates following the formation of a multidisciplinary diabetic foot care team [19]. Comparing the pre-integration (n = 137) and post-integration (n = 437) periods, Yesil and colleagues noted a significant decrease in overall amputation rates (20.4% vs. 12.6%) following the formation of the multidisciplinary team. Given that our tertiary care center has not yet developed an integrated service, we were not able to investigate any change in our amputation rates.

While it is essential to acknowledge that the results obtained from our study may not be generalizable at a national level due to the unique dynamics and practices at our institution, the insights we have gained certainly offer a valuable template for other similar healthcare institutions. Indeed, the incorporation of podiatric surgeons within a vascular division boasts a multitude of compelling advantages that warrant careful consideration. First and foremost, this strategic integration paves the way for fruitful multidisciplinary collaboration, thereby facilitating a comprehensive approach to patient care that capitalizes on the expertise of both specialties. Secondly, it serves as a potent deterrent against potential departmental conflicts that may arise. Third, the incorporation of podiatric surgeons within the vascular division serves to optimize the allocation of resources and expertise. By enabling vascular surgeons to channel their focus predominantly towards vascular procedures, the division can further hone their proficiency in this critical area, ultimately benefiting patient outcomes. Fourth, this integration preserves and bolsters podiatric revenue streams within the division, ensuring a sustainable financial model that supports the broader goals of the healthcare institution. Fifth, this collaborative approach extends beyond the confines of acute surgical interventions. It empowers podiatrists to leverage their extensive foot and ankle expertise not only in moments of immediate surgical need but also in the setting of prevention and long-term management of foot care for patients with vascular disease. This multidisciplinary approach not only enhances patient care but also contributes to the ongoing wellbeing and overall quality of life.

While this study yields valuable insights, it is crucial to consider the context of several inherent limitations that warrant attention. First, it is important to recognize that our study is retrospective in nature, which inherently introduces the possibility of bias. Second, the identification of all procedures within our study hinged on the meticulous review of institutional EMRs, a process that relies heavily on accurate coding and the completeness of documentation within operative reports. Any discrepancies or inaccuracies in these records could potentially impact the precision of our findings. Third, our dataset is inherently procedure-based, and as such, it lacks comprehensive information regarding non-surgical aspects of patient care, including but not limited to consultations, clinic visits, and any long-term follow-up care. These non-surgical facets are integral components of patient management, and their omission from our dataset is a noteworthy limitation. Fourth, it is essential to acknowledge that our study did not collect data pertaining to post-surgical outcomes, as mentioned above. This deliberate exclusion was made to remain within the scope of the study’s objectives; however, it is a limitation that should be considered when interpreting the broader implications of our findings. Despite these limitations, our study provides a granular and valuable contribution to the evolving body of literature concerning the integration of vascular–podiatric patient care. By recognizing these limitations, we aim to foster transparency in our research methodology and findings. The insights we have gained, while not without constraints, contribute to the ongoing dialogue surrounding the optimization of patient care through interdisciplinary collaboration.

## 5. Conclusions

In scenarios where podiatrists are not seamlessly integrated within a vascular surgical division, vascular surgeons may inherit clinical responsibilities inherent to podiatric diseases. They may find themselves shouldering a multifaceted role that encompasses an array of podiatric tasks, including consultations, surgical interventions, postoperative care, and long-term follow-up. While vascular surgeons may possess the requisite technical acumen to perform these podiatric procedures, it is imperative to acknowledge that specialized podiatric training in the areas of lower extremity biomechanics and long-term patient care is essential for optimizing patient outcomes. By recognizing the unique strengths and expertise that podiatrists bring to the table, this allows for a more comprehensive approach to patient care. In conclusion, our study underscores the potential for integrating podiatric surgeons within a vascular surgical division, presenting opportunities for collaboration and enhanced patient care.

## Figures and Tables

**Table 1:** Total podiatry procedures performed by vascular surgeons.

Category	N	(%)
Total procedures	1102	
*Primary procedure*	726	(65.9%)
*Additional procedure*	376	(34.1%)
Study year		
*2018*	148	(13.4%)
*2019*	179	(16.2%)
*2020*	266	(24.1%)
*2021*	276	(25.0%)
*2022*	233	(21.1%)

**Table 2:** Timing of podiatry procedures performed by vascular surgeons.

Category	N	(%)
Case type		
*Elective*	796	(72.2%)
*Urgent*	278	(25.2%)
*Emergent*	28	(2.5%)
After hours	163	(14.8%)
Holiday/weekend	133	(12.1%)
Day type		
*Weekday*	981	(89.0%)
*Weekend*	121	(11.0%)

After-hours cases are defined as procedures with start times before 0600 or after 1800.

**Table 3:** All podiatry procedures performed as primary procedures.

CPT	N	(%)	wRVU/Case	Total wRVU	Description
11061	1	(0.1%)	2.45	2.45	Incision and drainage of abscess, ankle/foot/toe
10180	4	(0.6%)	2.30	9.20	Incision and drainage of complex postoperative wound infection, ankle/foot/toe
11010	11	(1.5%)	4.19	46.09	Debridement at site of open fracture/dislocation, foot
11042	67	(9.2%)	1.01	67.67	Debridement, subcutaneous tissue, ankle/foot/toe
11043	141	(19.4%)	2.70	380.70	Debridement, muscle and/or fascia, ankle/foot/toe
28800	4	(0.6%)	8.79	35.16	Foot amputation, midtarsal
28805	173	(23.8%)	12.71	2198.83	Foot amputation, transmetatarsal
28810	118	(16.3%)	6.64	783.52	Toe amputation, metatarsal
28820	139	(19.1%)	3.51	487.89	Toe amputation, metatarsophalangeal joint
28825	68	(9.4%)	3.41	231.88	Toe amputation, interphalangeal joint
Total	**726**	**(100.0%)**		**4243.39**	

Abbreviations used: CPT, current procedural terminology; wRVU, work relative value unit.

**Table 4:** All podiatry procedures performed as additional procedures.

CPT	N	(%)	wRVU/Case	Total wRVU	Description
11010	7	(1.9%)	4.19	29.33	Debridement at site of open fracture/dislocation, foot
11012	13	(3.5%)	6.87	89.31	Debridement at site of open fracture/dislocation, leg
11042	47	(12.5%)	1.01	47.47	Debridement, subcutaneous tissue, ankle/foot/toe
11043	37	(9.8%)	2.70	99.90	Debridement, muscle and/or fascia, ankle/foot/toe
11044	1	(0.3%)	4.10	4.10	Debridement, bone, ankle/foot/toe
13160	3	(0.8%)	12.04	36.12	Secondary closure of surgical wound, ankle/foot/toe
15100	1	(0.3%)	9.90	9.90	Split-thickness skin graft, leg
15120	1	(0.3%)	10.15	10.15	Split-thickness skin graft, ankle/foot/toe
28005	65	(17.3%)	12.71	826.15	Foot amputation, transmetatarsal
28808	3	(0.8%)	4.59	13.77	Fasciotomy, foot and/or toe
28810	80	(21.3%)	6.64	531.20	Toe amputation, metatarsal
28820	83	(22.1%)	3.51	291.33	Toe amputation, metatarsophalangeal joint
28825	35	(9.3%)	3.41	119.35	Toe amputation, interphalangeal joint
Total	**376**	**(100.0%)**		**2108.08**	

Abbreviations used: CPT, current procedural terminology; wRVU, work relative value unit.

## Data Availability

Data may be shared upon request and institutional approval.

## Abbreviations

CPT	Current procedural terminology
EMR	Electronic medical records
IRB	Institutional Review Board
TMA	Transmetatarsal amputation
VAMC	Veterans Affairs Medical Center
wRVU	Work relative value unit
